# The Effect of Height Error on Performance of Propagation Phase-Based Metalens

**DOI:** 10.3390/mi15040540

**Published:** 2024-04-17

**Authors:** Yongxue Qiu, Liangui Deng, Yujie Zhan, Gongfa Li, Jianguo Guan

**Affiliations:** 1 State Key Laboratory of Advanced Technology for Materials Synthesis and Processing, School of Materials Science and Engineering, Wuhan University of Technology, Wuhan 430070, China; yxqiu1999@whut.edu.cn (Y.Q.); zhanyujie@whut.edu.cn (Y.Z.); guanjg@whut.edu.cn (J.G.); 2 School of Information Engineering, Wuhan University of Technology, Wuhan 430070, China; 3 Shenzhen Research Institute, Wuhan University, Shenzhen 518057, China; 4 The Key Laboratory for Metallurgical Equipment and Control Technology of Ministry of Education, Wuhan University of Science and Technology, Wuhan 430081, China; ligongfa@wust.edu.cn

**Keywords:** metalens, propagation phase, fabrication error, focusing efficiency

## Abstract

Metalenses, as a new type of planar optical device with flexible design, play an important role in miniaturized and integrated optical devices. Propagation phase-based metalenses, known for their low loss and extensive design flexibility, are widely utilized in optical imaging and optical communication. However, fabrication errors introduced by thin-film deposition and etching processes inevitably result in variations in the height of the metalens structure, leading to the fabricated devices not performing as expected. Here, we introduce a reflective TiO_2_ metalens based on the propagation phase. Then, the relationship between the height variation and the performance of the metalens is explored by using the maximum phase error. Our results reveal that the height error of the unit structure affects the phase rather than the amplitude. The focusing efficiency of our metalens exhibits robustness to structural variations, with only a 5% decrease in focusing efficiency when the height varies within ±8% of the range. The contents discussed in this paper provide theoretical guidance for the unit design of the propagation phase-based metalens and the determination of its allowable fabrication error range, which is of great significance for low-cost and high-efficiency manufacturing.

## 1. Introduction

A metasurface, a kind of two-dimensional metamaterial, is composed of subwavelength-resolved meta-atoms that can be designed to enable multidimensional manipulation of the polarization, phase, and amplitude of the optical field [1,2,3,4,5,6]. By controlling the geometry, orientation, and composition of these meta-atoms, the metasurface can be used in a variety of applications, such as optical imaging, information processing, energy harvesting, and optical sensing [7,8,9,10,11,12,13,14,15,16]. As a typical application of a metasurface, metalenses provide an effective technical way to reduce the load of traditional optical systems, realizing the integration and versatility of optical systems. Compared to plasma resonance-based metalenses, dielectric metalenses are widely used because of their wider operating frequency band and low loss. And propagation phase-based dielectric metalenses with polarization-insensitive properties have been widely studied due to their ability to achieve higher efficiency and wider angular coverage [17,18,19,20,21,22]. However, the practical application of a metalens depends not only on the initial design but also on the processing preparation. Despite significant advancements in design theory enabling the creation of highly efficient metalenses, the introduction of fabrication errors during manufacturing has often led to final device performances falling short of expectations [23,24,25,26,27]. In recent years, some researchers have delved into the effects of sidewall tilting, lateral dimension variation, and process defects of structural units on the performance of metalenses [28,29]. They have discovered that sidewall tilting leads to the proximity of neighboring structures, resulting in stronger structural resonance, which affects the efficiency of the metalens. Changes in lateral dimensions contribute to efficiency loss but have a smaller effect on beam quality. In contrast, the absence of structural units has the least effect on the performance of the metalens. Additionally, other researchers have analyzed the impact of fabrication errors on the focusing efficiency of metalenses in the infrared band [30,31,32]. These metalenses are robust to minor variations in structural units due to the large characteristic dimensions of the structural units in this band. However, metalenses operating in the visible to near-infrared wavelength bands encounter greater challenges due to their smaller structural dimensions (minimum feature size of less than one hundred nanometers) and larger aspect ratios. Therefore, it is essential to explore the influence of fabrication errors in propagation phase-based metalenses in the visible to near-infrared wavelength band.

Despite the rapid development of micro-nanofabrication technologies in the past few years [33,34,35,36], some problems still exist. For instance, the random distribution of deposited atoms during film deposition leads to non-uniformity in films, and the subsequent etching process is susceptible to loading effects, resulting in variations in etching depth [37,38,39,40,41]. However, the phase accumulation of metalenses based on the propagation phase is dependent on the height of the structure. Therefore, variations in the height of the metalens structure may directly affect the phase distribution of the optical field and the overall performance of the fabricated metalens. Hence, it is important to consider the effect of height error on the performance of metalenses, which can provide theoretical guidance for the cost-effective preparation of metalenses.

In this paper, to analyze the effect of height error on the focusing efficiency of a metalens, an analytical model for a reflective propagation phase-based metalens that enables line focusing was designed. The model is composed of a TiO_2_ nanobrick array with Ag substrate, working in the visible and near-infrared bands. Then, we introduce the height error into the TiO_2_ nanobricks, and analyze the effect of the height error on the amplitude and phase modulation. Moreover, by establishing a correspondence between the phase variation of the metalens and the focusing efficiency through the analytical method of maximum phase difference, we can quantify the effect of height error on the focusing efficiency of the metalens. The results provide detailed and effective theoretical guidance for the design and low-cost production of metalenses with controllable focusing efficiency.

## 2. Metalens Design

**Figure 1:**
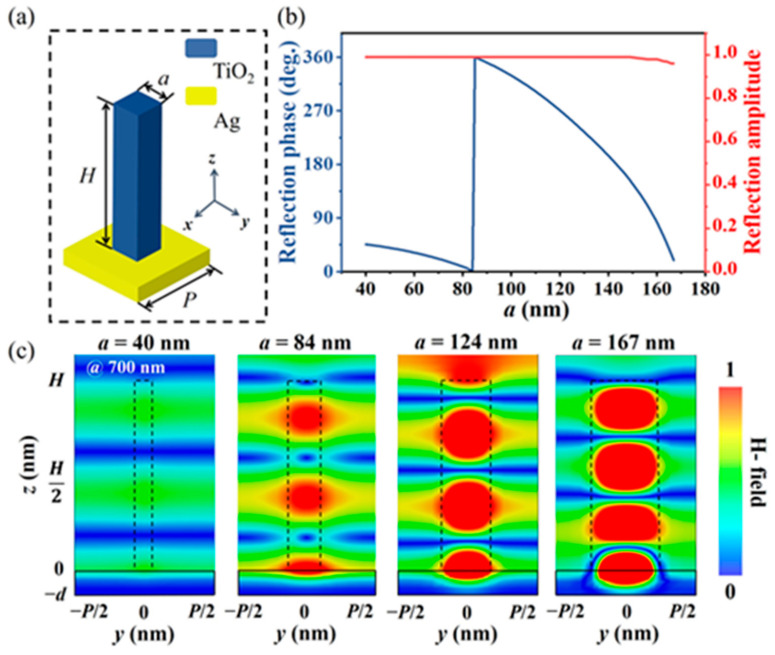
(**a**) Schematic of the basic element. (**b**) Reflection phase profile and amplitude of the unit cell with varying width at 700 nm wavelength. (**c**) The y–z section magnetic field distribution of the unit cell for different widths.

To explore the influence of metalens structure height on the focusing performance, the reflective basic unit shown in Figure 1a was designed. The typical unit is composed of a silver substrate and a titanium dioxide square column located on it. The refractive index of TiO_2_ is given by the dispersion equation [42], and the dielectric constant of Ag is calculated by the Drude model [43]. The thickness of the silver substrate d = 100 nm greater than the skin depth of visible light, which can effectively prevent the penetration of the incident field and provide additional phase shifting. Therefore, the reflective metalens may be more sensitive to fabrication errors than the transmissive. The structural units were simulated using CST Studio Suite (Dassault Systèmes simulation). The incident light is assumed to be x-polarized with a wavelength of 700 nm, propagating along the z-axis, with the electric and magnetic field vectors located in the x–y plane. The boundary conditions in both the x and y directions are unit cells, and in the z-direction, the boundary condition is open, and there is a vacuum layer of a certain thickness on top of the structure. A port is provided at the top of the vacuum layer, and the reflected light is collected at the port to obtain the phase delay and transmittance of the structure. To optimize the performance of the nanostructures, we swept the side lengths from 20 nm to 300 nm in steps of 5 nm. Finally, the height of the TiO_2_ square nanocolumn H = 800 nm with a period P = 350 nm can achieve full-phase coverage by changing the side length a (from 40 nm to 167 nm) [44,45], and in this process, the reflection amplitude of the unit is close to 1 (as shown in Figure 1b), meeting the basic design requirements for a reflective metalens [46,47]. To reveal the phase response mechanism of the meta-unit, as shown in Figure 1c, CST Microwave Studio was used to perform near-field simulation on the basic units with different side lengths by adding magnetic field monitors and extracting their magnetic field distribution maps. Figure 1c reveals that when the designed unit structure interacts with the normal incidence light field, the magnetic field energy can be localized inside the nanobricks, and the mutual coupling between adjacent structures can be suppressed. Therefore, a single TiO_2_ nanobrick can be regarded as an independent truncated waveguide for transmission phase modulation [48,49]. As the side length of the dielectric column increases (Figure 1c), the number of transmission envelopes in the dielectric column increases gradually, which is highly consistent with the unit simulation results in Figure 1b. In addition, considering the absorption of the light field caused by the plasma resonance between the titanium dioxide nanobricks and the silver substrate (as shown in the fourth figure in Figure 1c), it is further revealed that when the side length of the structure in Figure 1b is larger, the reflection amplitude is attenuated.

**Figure 2:**
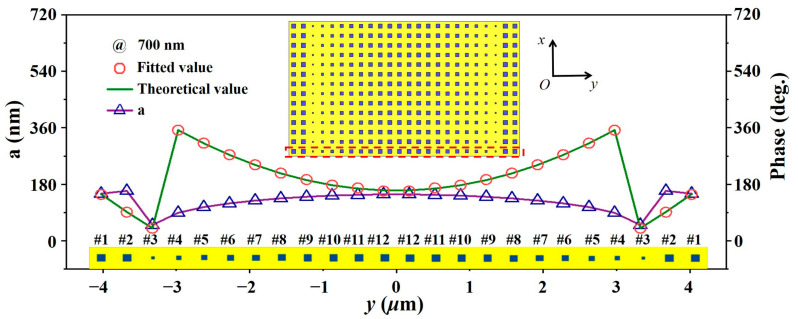
The side lengths “a” corresponding to the different positions of the metalens, and the corresponding theoretical and fitted phase profiles at the wavelength of 700 nm. Sketches of the corresponding meta-atoms are listed above the y-axis. The inset shows the metalens consisting of 24 × 14 unit cells. #1–#12 represent the 12 units selected.

Based on the above reflective basic structural unit, we designed a reflection-type line-focusing metalens, with a focal length of 11.62 μm, numerical aperture (NA) of 0.34, and size of 8.4 μm × 4.2 μm (as shown in Figure 2). To obtain a metalens with line-focusing characteristics, as shown in the inset in Figure 2, the phase distribution in the aperture field along the y direction must satisfy the following parabolic equation:

(1) $$\varphi =-k_{0}\left(\sqrt{y^{2}+f^{2}}-f\right)+∆\varphi ,$$

where *k*_0_ is the free space wave vector, *y* is the distance from the basic unit to the lens center, *f* represents the focal length, and ∆*φ* is the reference phase. Then, because the designed structure is a line-focused two-dimensional metalens, the desired metalens can be finally obtained by translating the nanostructures arranged in the y direction along the x direction. Commercial mathematical software (MATLAB, R2021b, MathWorks, Natick, MA, USA) was used to match the target phase to the phase library. In the metalens array simulation, the boundary conditions were set to open boundaries in all directions. The plane wave port was placed at 15 μm above the metalens array and a power flow monitor was added to collect the transmitted and reflected power flow intensity. The mesh was set as an adaptive mesh. Considering that the near-field coupling between elements can lead to unpredictable shifts in the phase delays obtained from the simulation of elements within the array, the reference phase ∆*φ* was swept parametrically here, which ranges from 0 to 350° in 10° steps. Each reference phase was substituted into Equation (1) to obtain the corresponding 12 side lengths. The selected side lengths are used for array and full-wave simulations sequentially. The focusing efficiencies of different metalens arrays were further calculated for comparison, and it was finally found that the metalens has the highest focusing efficiency when the reference phase is 160°. This indicates that the near-field coupling effect can be weakened by designing a reasonable reference phase, thus obtaining the best focusing performance. Through the above optimization process, the final edge length and phase distribution of the array are shown in Figure 2.

**Figure 3:**
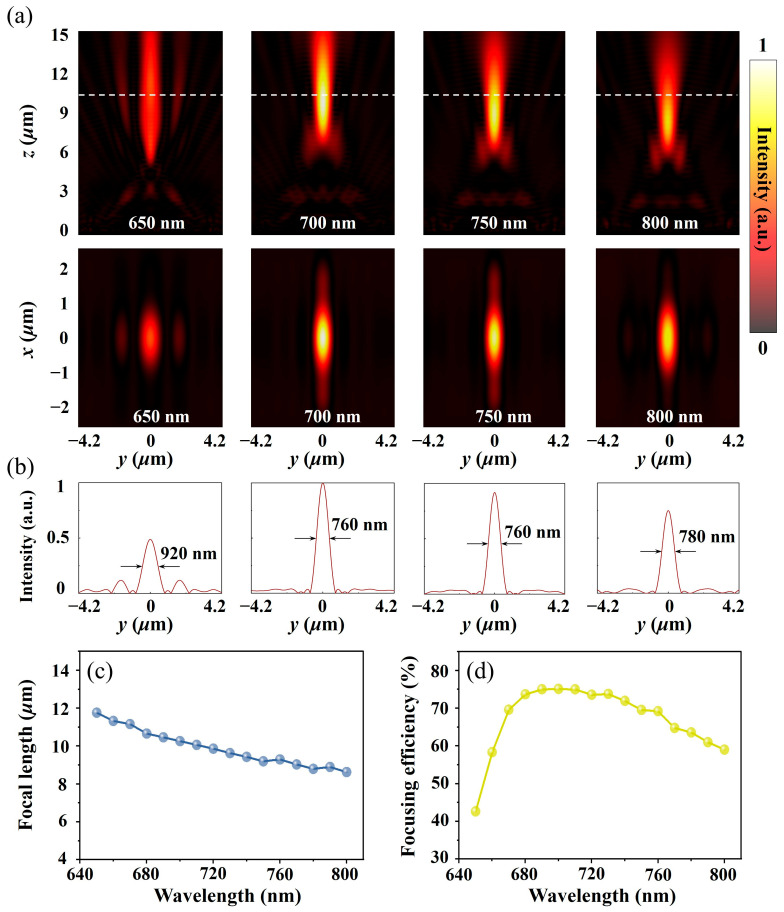
(**a**) Simulated power flow intensity distributions in the y–z plane (first line) for different incident wavelengths and in the x–y plane (second line) for different incident wavelengths at the designed focal length. The white dashed line indicates the position of the focal plane. (**b**) The normalized power flow intensity profile along the horizontal (y direction) line cuts through the center of the focal spot at selected wavelengths. Focal length (**c**) and focusing efficiency (**d**) as a function of the incident wavelength.

To show the focusing performance of the designed metalens, a full-wave metalens simulation was immediately carried out, and the power flow density distribution on the focal plane (y–z plane and x–y plane in Figure 3a) was extracted (in the simulation process, a plane wave was used as the excitation source, and the polarization was along the x direction). Figure 3a shows that when the wavelength of the normal incident light gradually increases from 650 nm to 800 nm, the corresponding focal length gradually decreases, which conforms to the dispersion characteristics of the light field. In addition, the second line of Figure 3a shows that the collection ability of the metalens toward the light wave increases first and then decreases and reaches the maximum at the preset wavelength of 700 nm. The attenuation of the focusing performance at short wavelengths occurs mainly because the working wavelength is equivalent to the size of the designed unit structure, which leads to the enhancement of the high-order diffraction effect and reduction in the controllable fundamental wave energy. This analysis is verified from the split focal spot at 650 nm (the leftmost graph of Figure 3b). The focusing attenuation caused by the increase in the wavelength can be attributed to the nonideal phase distribution (deviation from the ideal value designed in Formula (1)) caused by the unit phase dispersion. The focal spot size corresponding to different wavelengths is given in Figure 3b, and the focal spot size can laterally reflect the strength of the binding ability of the metalens toward space waves (the smaller the focal spot size is, the stronger the convergence ability), which is consistent with the previous analysis. Then, to further analyze the focusing characteristics of the designed metalens, the relationship between the focal length (Figure 3c), focusing efficiency (Figure 3d), and incident wavelength was obtained through numerical simulation. Focal length is defined as the distance from the energy maximum in the y–z plane to the surface of the metalens. Focusing efficiency is defined as the ratio of the reflected power flow collected by the metalens to the total incident power flow. From Figure 3d, the average focusing efficiency of the designed metalens in the wavelength range of 650 nm to 800 nm is approximately 67%, and the maximum focusing efficiency (75%) is obtained at the design wavelength of 700 nm. The overall structure shows good focusing ability. The change in focal length (Figure 3c) further illustrates that as the focal length deviates more from the design focal length, its focusing effect decreases more severely.

## 3. Error Analysis

**Figure 4:**
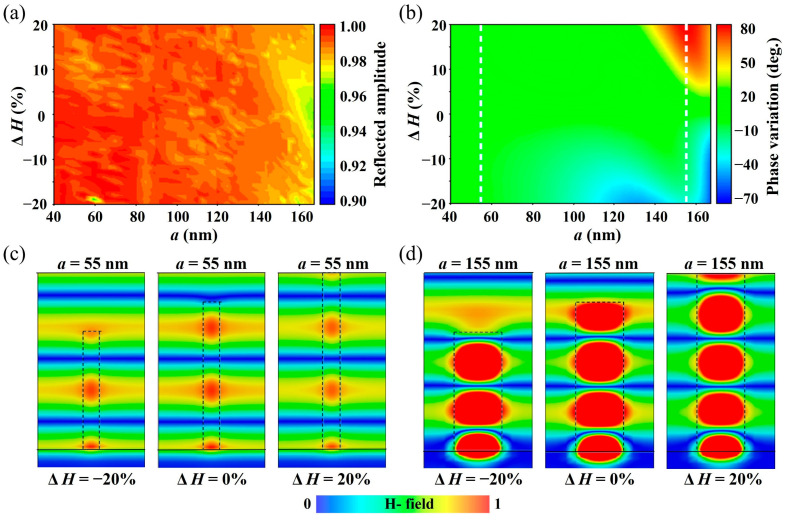
Reflection amplitude (**a**) and phase variation (**b**) as a function of the height variation (Δ*H*) with different widths a. Magnetic field distributions inside the basic element under different height variations (Δ*H*) when a = 55 nm (**c**) and a = 155 nm (**d**).

Up to this point, the line-focusing metalens as the analysis reference has been designed. Therefore, the influence of the height change on the focusing performance can be explicitly studied. For metalenses, the core factor affecting the focusing efficiency is the phase and amplitude distribution of the aperture plane. The requirements are as follows: the phase distribution satisfies Formula (1), and the amplitude is uniform and nearly lossless. Therefore, to analyze the influence of the change in the surface uniformity of the metalens on the above factors, it is necessary to first obtain the reflection amplitude change and phase shift caused by the change in the unit structure height. For this reason, for the basic unit shown in Figure 1, by changing the height of the TiO_2_ nanobricks, we respectively obtained a two-dimensional amplitude map (Figure 4a) and a phase variation map (Figure 4b) that changes with the side length. The simulation process uses 700 nm as the working wavelength, and the height variations in the TiO_2_ nanobricks are controlled within ±20%. Figure 4a indicates that the change in the TiO_2_ nanobrick height has little impact on the reflection amplitude of the overall unit structure, so this is not the main factor leading to efficiency degradation. In contrast, the change in the structural unit height has a more significant effect on the reflection phase delay. As shown in Figure 4b, with the increase in the height of the TiO_2_ nanobricks, the corresponding phase shift deviates from the ideal value more. This phenomenon can be explained by analyzing the principle of truncated waveguide phase shift. Based on the foregoing analysis, the designed meta-units can be regarded as a truncated waveguide, and the transmission phase change can be expressed as

(2) $$∆\varphi =\frac{2\pi }{\lambda }n_{eff}∆H$$

where *λ* is the wavelength of the incident light, *n_eff_* is the effective refractive index of the structural unit, and ∆*H* is the height variations in the dielectric column structure. Formula (2) shows that for TiO_2_ nanobricks with the same side length (i.e., the same *n_eff_*), a larger ∆*H* can undoubtedly lead to a larger phase shift, which is highly consistent with the phenomenon shown in Figure 4b. To further visualize the physical principle of the above reflection phase shift changing with the structure height, as a comparison, the distributions of the internal magnetic field corresponding to the height variations in the TiO_2_ nanobricks when a = 55 nm (Figure 4c) and 155 nm (Figure 4d) are given. The magnetic field envelope number is positively correlated with the height of the dielectric column. In addition, considering the positive correlation between *n_eff_* and the duty cycle of the titanium oxide nanobricks, when ∆*H* is the same, ∆*φ* tends to increase as the side length of the nanobricks increases. This conclusion can be confirmed by comparing Figure 4c,d. Figure 4b shows that when a = 155 nm and the height of the nanobricks has a negative deviation, there is no expected phase shift theoretically, mainly because the appearance of Fabry–Pérot resonance alleviates the phase shift caused by the waveguide mode [28,50]. Specifically, the side length a affects the shape and size of the nanobricks, which in turn affects the effective refractive index of the structural unit. Structural units with larger side lengths lead to a larger effective refractive index, which has a greater impact on the phase. The height H of the nanobrick directly affects the optical path of light through the structure, so a greater height means that the light needs to travel through a thicker medium, which leads to a greater phase transition. Therefore, the side length a and the height H affect the phase in different ways, and it is not possible to specify which parameter has a greater effect on the phase. However, it is worth noting that smaller side lengths are less sensitive to changes in height. Therefore, in the design of a metalens, it may be more desirable to choose structural units with smaller side lengths, as this can reduce the effect of the error on the phase to some extent and improve the performance and accuracy of the metalens.

**Figure 5:**
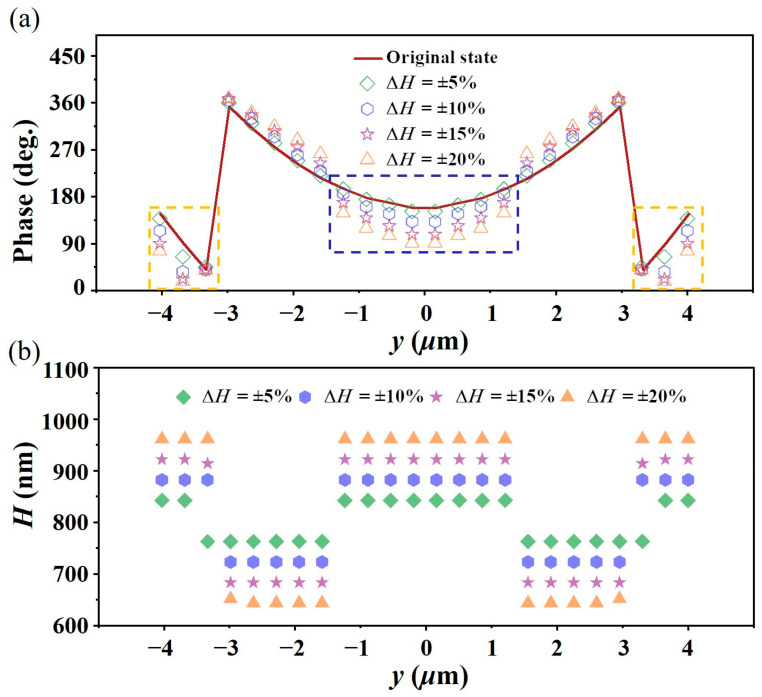
(**a**) Phase variation with respect to the original phase at different height variations for different unit cells on the y-axis. (**b**) Selected height in the range of different height variations for different unit cells on the y-axis.

To analyze the influence of the array height variation on the focusing performance of the initially designed metalens, different height errors in composition units #1–#12 were introduced. Considering the different array height variations, the dielectric column with the maximum reflection phase difference from the ideal phase value was selected to conduct array formation and full-wave simulation again. In this process, the side length of the TiO_2_ nanobricks at the corresponding position remains unchanged. As shown in Figure 5a, when Δ*H* = ±5%, ±10%, ±15%, and ±20%, based on the aperture field phase distribution along the y direction of the metalens, the unit reflection phase offset gradually increases with the increase in the upper limit of the height variations, which is consistent with the above analysis. Table 1 shows the specific phase offset. The phase offset is the maximum value of the phase deviation of the nanobricks from the designed ideal phase at the maximum height error. Figure 5b shows the selected 12 structural unit heights, with maximum height errors of ±5%, ±10%, ±15%, and ±20%. For example, at a maximum height error of ±10% (indicated by the blue hexagon), the height of the distributed nanobricks on the y-axis corresponds to the vertical coordinate of the blue hexagon. It is evident that the maximum height variation basically corresponds to the highest degree of phase variation, which is consistent with the principle of the propagation phase.

**Figure 6:**
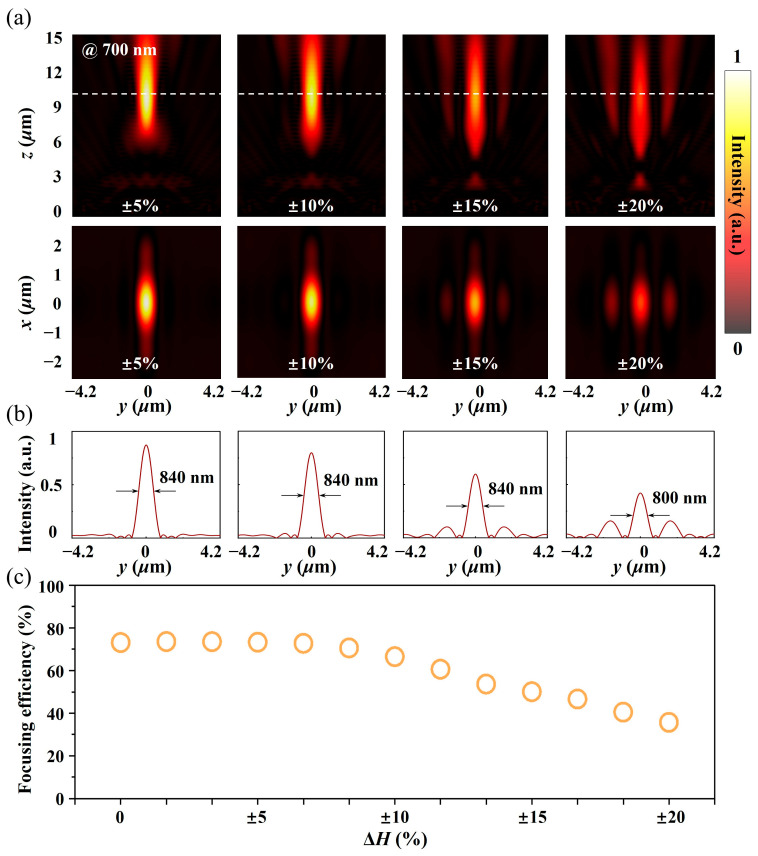
(**a**) Simulated power flow intensity distribution in the y–z plane (first line) and x–y plane (second line) at different height variations with a working wavelength of 700 nm. The white dashed line indicates the position of the focal plane. (**b**) The normalized power flow intensity profile along the horizontal (y) line cuts through the center of the focal spot at different height variations with a working wavelength of 700 nm. (**c**) Focusing efficiency as a function of height variation error.

A full-wave simulation was carried out for the reconstructed metalens, and the power flow distribution on the focal plane (operating wavelength of 700 nm) was extracted, as shown in Figure 6a,b. The distribution of the power flow density on the y–z plane in Figure 6a reveals that with the deterioration of surface flatness, the power flow density at the focal spot gradually decreases, accompanied by the appearance of sidelobes, while the focal length exhibits almost no change in this process (dotted line in Figure 6a). The attenuation of the focusing efficiency can be attributed to the nonideal phase distribution, while the appearance of sidelobes can be concluded from the phase distribution in Figure 5a. The phase distribution gradient at the center of the array (in the blue dotted box) and at the edge (in the yellow dotted box) gradually increases with the increase in the unit height change, while the change in the phase gradient can directly affect the outgoing direction of the reflected light field, leading to stray focusing energy. The power flow density distribution on the x–y focal plane in Figure 6a shows the same change rule as that on the y–z focal plane. Figure 6b shows that compared with the ideal array, the focal spot size gradually diffuses with the increase in the height error, which demonstrates that the field convergence ability of the metalens gradually degrades in this process. In addition, the enhancement of the sidelobe energy further leads to the attenuation of the focusing efficiency.

To further reveal the influence of the height variations in the TiO_2_ nanobricks on the focusing efficiency of the metalens and guide the processing of low-cost micro/nano-devices, the linear relationship between the height variations in the TiO_2_ nanobricks and the focusing efficiency was obtained by numerical calculation, as shown in Figure 6c. Figure 6c shows that the focusing efficiency of the metalens first decreases steadily and then decays rapidly with the increase in the height error. The inflection point appears at Δ*H* = ±8%, and the focusing efficiency attenuation is approximately 5%. This indicates that the height variation of the metalens structure is robust to the focusing efficiency of the metalens.

## 4. Conclusions

In summary, based on the designed analytical model of a TiO_2_/Ag reflective propagation phase-based metalens in the visible to near-infrared band, the influence of the height variations in the basic unit on the amplitude and phase distribution of the aperture field was explored in detail. Through comprehensive comparative analysis, it was found that height variations hardly affect the amplitude, and the primary determinant of focusing efficiency is the phase perturbation within the aperture field. Notably, structural units with shorter side lengths exhibit lower sensitivity to height fluctuations, which is attributed to the relatively little phase accumulation when light waves pass through them.

Furthermore, the analytical method of maximum phase difference was employed to elucidate the fundamental relationship between height variations and the focusing efficiency. The simulation result shows that the metalens focusing efficiency was demonstrated to be robust to height deviations. Specifically, within a ±5% range of height variation, the focusing efficiency remains relatively stable. Additionally, we established an allowable range of height error for the TiO_2_ nanobricks at ±8%, with a 5% reduction in focusing efficiency serving as the prescribed reference threshold. Although multiple processing processes are involved in the whole metalens preparation process in addition to the height error, and different processing processes may bring different fabrication errors—such as transverse dimensional variations caused by the proximity effect of e-beam exposure, conical nanostructures, and missing nanostructures caused by transverse etching of the etching process, etc.—the maximum phase difference method used in this paper is also applicable to the analysis of these different fabrication errors and can provide guidance for the optimization of the preparation process based on the results of the maximum phase difference method. In summary, this study can provide theoretical guidance not only for high-quality propagation phase-based metalens processing, but also for the fabrication error control of the whole micro/nano-processing of other optical devices, such as holographic plates and optical waveguides.

## Figures and Tables

**Table 1:** The maximum phase difference of different meta-units with different height variations.

	ΔH	±5%	±10%	±15%	±20%
No.					
#1		−12.29	−35.72	−60.34	−74.93
#2		−29.57	−58.46	−72.66	−77.10
#3		1.63	−3.23	−3.76	−4.25
#4		5.93	10.96	13.80	14.50
#5		7.88	17.39	24.61	28.27
#6		6.96	19.05	30.77	38.91
#7		4.99	16.28	31.26	44.78
#8		2.59	11.57	27.09	44.99
#9		−2.59	−11.40	−28.60	−48.85
#10		−5.13	−18.92	−40.61	−60.65
#11		−6.89	−23.68	−47.08	−65.93
#12		−9.27	−29.17	−53.61	−70.63

## Data Availability

The data presented in this study are available on request from the corresponding author.
